# Validation of Prognostic Circulating Cell-Free RNA Biomarkers *HPGD*, *PACS1*, and *TDP2* in Colorectal Cancer Through TaqMan qPCR and Correlation Analysis

**DOI:** 10.3390/cimb47070508

**Published:** 2025-07-02

**Authors:** Chau Ming Kan, Xiao Meng Pei, Simon Siu Man Ng, Wing Wa Leung, Yee Ni Wong, Hennie Yuk-Lin Cheng, William Chi Shing Cho, Hin Fung Tsang, Sze Chuen Cesar Wong

**Affiliations:** 1 Department of Applied Biology & Chemical Technology, The Hong Kong Polytechnic University, Hong Kong SAR, China; kantrevor@gmail.com (C.M.K.); xiaomeng2019.pei@polyu.edu.hk (X.M.P.); hennie.cheng@polyu.edu.hk (H.Y.-L.C.); andy_thf@yahoo.com.hk (H.F.T.); 2 Department of Surgery, Faculty of Medicine, The Chinese University of Hong Kong, Hong Kong SAR, China; simonng@surgery.cuhk.edu.hk (S.S.M.N.); leungww@surgery.cuhk.edu.hk (W.W.L.); cherry@surgery.cuhk.edu.hk (Y.N.W.); 3 Department of Clinical Oncology, Queen Elizabeth Hospital, Kowloon, Hong Kong SAR, China; williamcscho@gmail.com

**Keywords:** colorectal cancer (CRC), Taqman quantitative PCR (qPCR), circulating cell-free RNAs (cfRNAs), *HPGD*, *TDP2*, *PACS1*, prognostic biomarkers, minimal residual disease (MRD)

## Abstract

Circulating cell-free RNAs (cfRNAs) have emerged as promising non-invasive biomarkers for colorectal cancer (CRC), offering insights into the disease’s prognosis. This study investigates the prognostic significance of the specific cfRNA biomarkers *HPGD*, *PACS1*, and *TDP2* by employing the Taqman quantitative PCR (qPCR) to evaluate their expression levels in a cohort of 52 CRC patients. The methodology involved a robust statistical analysis to assess correlations between cfRNA levels and clinical parameters, including survival rates and recurrence incidences. Findings revealed a significant upregulation in the expression of *HPGD* and *PACS1*, while *TDP2* displayed varying results, indicating a complex role in disease dynamics. Notably, lower expression levels of *HPGD* were associated with reduced survival, suggesting its potential as a negative prognostic indicator. Conversely, *TDP2* levels correlated strongly with increased risks of recurrence, highlighting its clinical relevance in monitoring disease progression. Overall, this study elucidates the intricate interplay between these cfRNAs in the CRC prognosis. The results advocate for further exploratory studies to validate *PACS1*’s potential as a prognostic marker and reinforce the clinical significance of *HPGD* and *TDP2* in the context of CRC management, positioning them as vital elements in the landscape of molecular oncology.

## 1. Introduction

Colorectal cancer (CRC) remains a significant global health burden and is the third most common cancer diagnosis, as well as the second leading cause of cancer-related deaths worldwide [1]. Despite significant advances in surgical techniques and adjuvant treatments, CRC patient outcomes remain compromised by high recurrence rates and frequent late-stage diagnoses [2]. Improving patient outcomes lies in the early identification of minimal residual disease (MRD) following operations, enabling personalized treatment strategies and improving survival rates [3]. Traditional methodologies for detecting MRD, such as imaging and tissue biopsies, are invasive, expensive, and often lack the sensitivity needed for timely intervention [4]. These limitations underscore an urgent requirement for the development of non-invasive, reliable biomarkers capable of predicting the CRC recurrence and prognosis [5]. For instance, Guardant has promoted a completely tissue-free test that detect circulating tumor DNA for monitoring the recurrence and MRN assessment in early stages of breast, colorectal, and lung cancer [6].

Recent advancements have highlighted the promise of circulating cell-free RNAs (cfRNAs) in plasma as non-invasive biomarkers for cancer detection and prognosis in CRC. cfRNAs, which include both messenger RNAs (mRNAs) and non-coding RNAs, are released into the bloodstream by various cells, notably tumor cells, and thereby offer insight into the molecular dynamics of the tumor microenvironment. Building on this potential, our previous study identified three significant cfRNA biomarkers comprising *HPGD* (hydroxyprostaglandin dehydrogenase), *PACS1* (Phosphofurin Acidic Cluster Sorting Protein 1), and *TDP2* (tyrosyl-DNA phosphodiesterase 2) via the RNA sequencing (RNA-seq) of plasma samples from a small cohort of CRC patients. This signature demonstrated consistent expression changes before and after tumor-ablative operations in plasma and tissue samples, with a further validation using TCGA CRC datasets and single-cell RNA-seq for cell type deconvolution, which was linked to the presence of MRD, suggesting its utility in tracking the disease progression [7]. However, the limited sample size and reliance on RNA-seq, a method not yet widely adopted in clinical diagnostics due to its complexity and cost, underscore the need for further validation using more accessible and clinically feasible techniques [7].

This expanded study seeks to validate the prognostic significance of the *HPGD*, *PACS1*, and *TDP2* cfRNA signature in a larger cohort of 52 CRC patients, using the Taqman quantitative PCR (qPCR) as the primary method for the gene expression analysis. The Taqman qPCR is a highly sensitive, specific, and reproducible technique, widely utilized in clinical settings for its ability to detect low-abundance transcripts with precision. By examining plasma samples collected pre- and post-operation, this study seeks to verify the expression patterns of these genes and evaluate their links to key clinical outcomes, including the tumor stage, recurrence risk, and patient survival. Building on the foundational work of Jin et al. (2023), this research enhances the practical utility of cfRNA-based biomarkers by employing a more cost-effective and clinically adaptable method compared to RNA-seq [7].

The objective of this study is to validate the significance of three key biomarkers within a substantially larger patient cohort and methodically correlate their expression profiles with relevant clinical parameters; this research aims to establish a comprehensive and reliable tool that could substantially enhance CRC prognostication. Transitioning from RNA sequencing to the TaqMan qPCR signifies a strategic advancement in the endeavor to translate cutting-edge genomic research into practical diagnostic applications. This shift not only underscores the necessity for actionable diagnostics in clinical settings but also raises prospects for the improved management of CRC through timely detection and intervention, ultimately fostering enhanced patient outcomes in this prevalent malignancy.

## 2. Materials and Methods

### 2.1. Patient Cohort and Sample Collection

This study enrolled 52 patients diagnosed with CRC, who were scheduled for tumor-ablative operation at Prince of Wales Hospital (PWH) between May 2020 and January 2025. Patients were included if they had histologically confirmed CRC, provided pre- and post-operative plasma samples, and provided informed consent. Patients with hereditary CRC and inflammatory bowel disease were excluded in this study. The joint Chinese University of Hong Kong–New Territories East Cluster Clinical Research Ethics Committee (CUHK-NTEC CREC; Ref No: 2019.542) approved this study, and all participants provided written consent. Each patient was invited to donate blood (pre-operation on the day before operation and post-operation on the 5th–7th day after operation) for research purposes with written informed consent before the operation. Plasma isolation was conducted within 3 h of collecting anti-coagulated blood using the VACUETTE^®^ TUBE 2 mL K2E K2EDTA (Cat#454024, Greiner Bio-one, Kremsmünster, Austria). The blood was first centrifuged at 1600× *g* for 10 min at 4 °C. The upper plasma layer was carefully collected into a new tube without disturbing the buffy coat, then re-centrifuged at 16,000× *g* for 10 min at 4 °C to eliminate any remaining cell pellet [7,8]. The plasma was then harvested and preserved with 2 mL TRIzol™ LS Reagent (Cat#10296028, Thermo Fisher Scientific, Waltham, MA, USA) before being stored at −80 °C. The extraction of cfRNA from blood was performed following the methodology outlined in the prior study by Jin et al. (2023) [7].

### 2.2. RNA Extraction and Reverse Transcription

Cell-free RNA (cfRNA) was extracted from 2 to 3 mL of plasma using the miRNeasy Serum/Plasma Kit (Cat# 217204, Qiagen, Hilden, Germany) per the manufacturer’s instructions. The RNA quantity was measured by Qubit™ RNA High Sensitivity (HS) (Cat# Q32852, Invitrogen™, Waltham, MA, USA). Reverse transcription reactions were conducted as per the manufacturer’s guidelines using PrimeScript RT Master Mix (Cat# RR036B, Takara Bio Inc., Shiga, Japan) in 10 µL reaction volumes. Taqman qPCRs were performed using the TaqMan Fast Advanced Master mix (Cat# 4444557, Thermo Fisher Scientific, USA) in Stepone Plus Real-Time PCR System (Thermo Fisher Scientific) in a 9 μL reaction volume according to the manufacturer’s instructions. Commercially available Taqman Gene Expression Assays (Applied Biosystems, Singapore) were used, *HPGD* (Hs00960591_m1), *PACS1* (Hs01555555_g1), and *TDP2* (Hs01099017_m1), with *GAPDH* (Hs02786624_g1) as the reference gene for normalization (Table 1).

### 2.3. Statistical Analysis

Statistical analyses were conducted using JASP (version 0.19.3, JASP Team, 2024) to evaluate gene expression changes in *HPGD*, *PACS1*, and *TDP2* and their associations with clinical outcomes in 52 CRC patients, with all analyses performed on log_2_ fold change values derived from Taqman qPCR data using the ΔΔCt method, where ΔCt was calculated as the difference between the cycle threshold (Ct) of the target gene and the reference gene (*GAPDH*) [ΔCt = Ct(target) − Ct(*GAPDH*)], and ΔΔCt was computed as the difference between post-operation and pre-operation ΔCt values [ΔΔCt = ΔCt (post-operation) − ΔCt(pre-operation)], and log_2_ fold change was derived as −ΔΔCt, equivalent to log_2_(2^(−ΔΔCt)), to assess expression changes post-operation relative to pre-operation, with negative values indicating downregulation and positive values indicating upregulation; one-sample *t*-tests in JASP’s T-Tests module were used to determine if log_2_ fold change values significantly differed from zero (indicating no expression change), while correlation analyses in the Regression module, using Pearson’s r for normally distributed continuous variables, assessed associations between gene expression (log_2_ fold change) and clinical variables (e.g., tumor stage, recurrence, etc.), with scatter plots generated in the Microsoft Excel to visualize significant associations, setting statistical significance at *p* < 0.05. Any ambiguous results were omitted from the analysis of each biomarker by employing criteria for the removal of extreme or outlier values that could skew results, such as greater than two standard deviations from the mean.

## 3. Results

### 3.1. The Evaluation of the Taqman qPCR Results Match with the RNA Sequencing from a Previous Study

**Figure 1:**
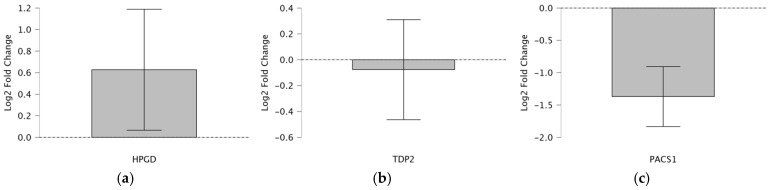
Shows the log_2_ fold change in the *TDP2* cfRNA expression (post-operative vs. pre-operative) in CRC patients. The gray bar represents the mean log_2_ fold change. The error bar (vertical line) shows the variability, likely the 95% confidence interval. Using a one-sample *t*-test with the test value set to 0, this suggests that (**a**) the increase in the *HPGD* expression is statistically significant (*p* < 0.05), as the confidence interval does not include 0, indicating a consistent upregulation post-operation. (**b**) The *TDP2* expression is not statistically significant (*p* > 0.05), as the confidence interval includes 0, meaning there is no strong evidence of a consistent up- or downregulation post-operation. (**c**) The decrease in the *PACS1* expression is statistically significant (*p* < 0.05), as the confidence interval does not include 0, indicating a consistent downregulation post-operation.

To evaluate the consistency of the Taqman qPCR results with the RNA-seqdata from a previous study [7] we applied a one-sample *t*-test (test value = 0) to assess the log_2_ fold changes in the cfRNA expression of *HPGD*, *PACS1*, and *TDP2* in CRC patients (Figure 1a–c). The analysis leverages a table summarizing *t*-test statistics for 52 patients and a box plot visualizing *HPGD*, *PACS1*, and *TDP2*’s log_2_ fold change pre- and post-operation. The one-sample *t*-test determines whether the mean log_2_ fold changes significantly deviate from 0, indicating up- or downregulation, while comparing the directional regulation (up or down) between the Taqman qPCR and RNA-seq to validate the reliability of Taqman as a more clinically feasible alternative to RNA-seq (Table 2).

HPGD shows a significant upregulation with a log_2_ fold change of 2.254 (*p* = 0.029, *t* = 2.254, *df* = 43), which is consistent across both Taqman and RNA-seq methods, indicating a robust increase in expression post-operation (fold change 2^2.254^ ≈ 4.77). In contrast, *TDP2* exhibits a non-significant downregulation via Taqman (log_2_ fold change = −0.398, *p* = 0.693, *t* = −0.398, *df* = 41; fold change 2^−0.398^ ≈ 0.76), despite the RNA-seq suggesting upregulation, highlighting a discrepancy possibly due to the methodological sensitivity or sample variation. *PACS1* demonstrates a significant downregulation (log_2_ fold change = −5.938, *p* < 0.001, *t* = −5.938, *df* = 48; fold change 2^−5.938^ ≈ 0.016), consistently observed in both Taqman and RNA-seq, suggesting a substantial decrease in the expression post-operation. These findings indicate varied expression patterns among the genes, with *HPGD* and *PACS1* showing clear directional changes, while TDP2’s regulation requires further investigation due to conflicting results.

### 3.2. Correlation Analysis of Biomarkers and Clinical Outcomes

**Figure 2:**
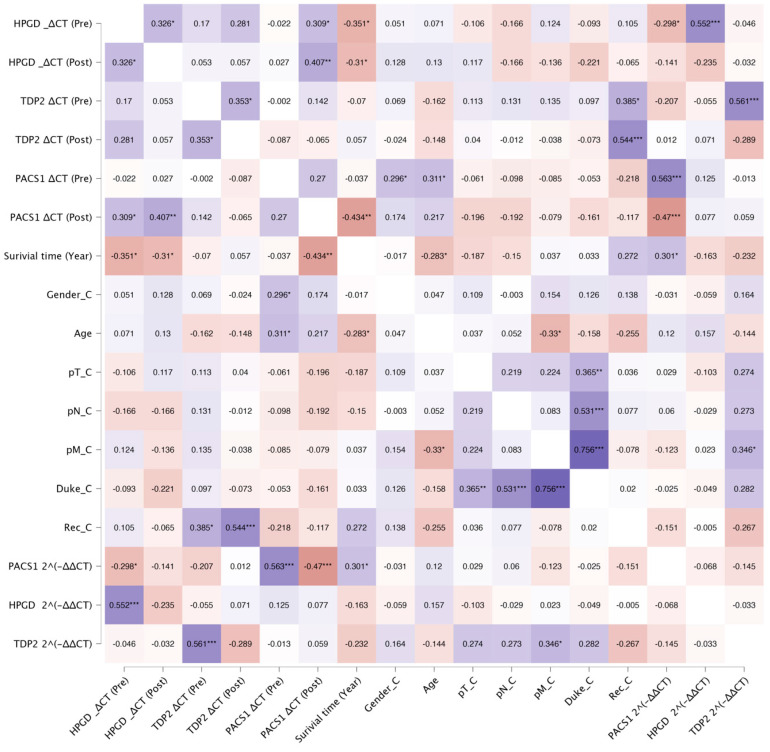
Correlation heatmap of biomarkers, survival, recurrence, and clinical variables. Color intensity represents Pearson’s correlation coefficient (r), with purple indicating positive correlations and red indicating negative correlations. Asterisks denote significant correlations (* *p* < 0.05, ** *p* < 0.05, and *** *p* < 0.005).

The correlation analysis revealed several significant associations between the biomarkers, survival outcomes, recurrence, and Duke stage in the studied cohort. Below, we summarize the key findings grouped by the biomarker and clinical variables (Figure 2).

### 3.3. HPGD

The relationship between the *HPGD* expression levels, quantified using the Taqman assay (ΔCT values), and patient survival was thoroughly analyzed.

**Figure 3:**
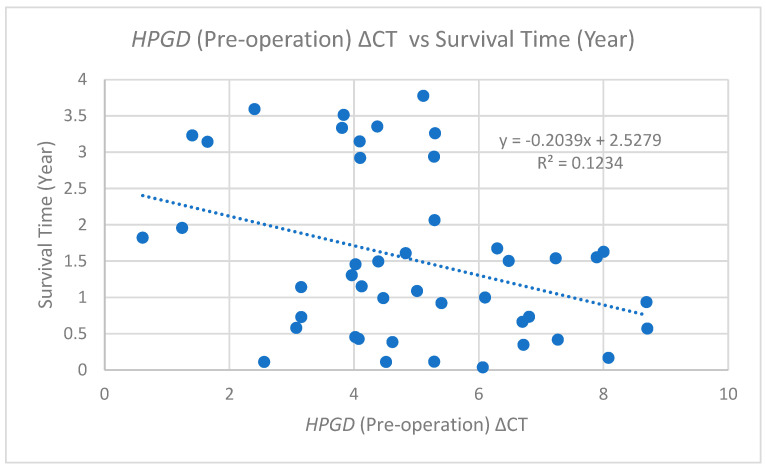
The scatter plot indicates this relationship through a dashed trend line with the equation (y = −0.2039x + 2.5279), yielding an R^2^ value of 0.1234.

A significant negative correlation was identified between the pre-operative ΔCT values and survival time (years). As shown in Figure 3, this suggests that higher pre-operative ΔCt values (lower *HPGD* expression) are associated with shorter survival times. However, the low R^2^ value (0.1234) indicates a weak correlation, meaning that the *HPGD* expression pre-operation explains only a small portion of the variability in the survival time.

**Figure 4:**
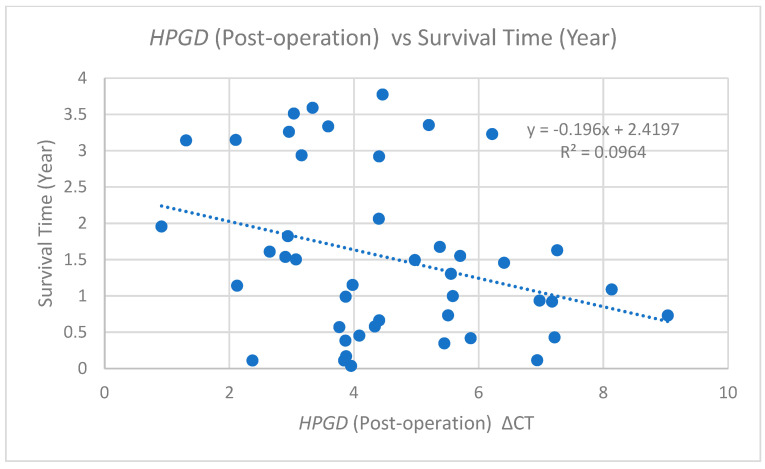
Scatter plots with linear regression lines showing the relationship between the *HPGD* expression and survival time (years) in post-operative cfRNA samples. For post-operative samples, the regression equation is y = −0.196x + 2.4197, with an R^2^ value of 0.0964.

Similarly, an additional analysis of post-operative ΔCT values relative to the survival time is depicted in Figure 4. This scatter plot illustrates a comparable negative correlation, represented by the trend line equation (y = −0.196x + 2.4197) and an R^2^ value of 0.0964. This correlation is slightly weaker than that observed for pre-operative values, suggesting that higher post-operative ΔCt values (lower *HPGD* expression) are associated with shorter survival times.

The alignment of the correlation trends between pre- and post-operative measurements reinforces the notion of *HPGD*’s potential as a prognostic biomarker. Specifically, these findings indicate that patients demonstrating a lower HPGD expression whether assessed pre-operatively or post-operatively may experience shorter survival times.

### 3.4. TDP2

The analysis of *TDP2* expression levels through the TaqMan quantitative PCR (qPCR) revealed significant associations with both recurrence rates and the ΔCT values of *TDP2*. Specifically, higher expression levels of *TDP2* were correlated with lower *TDP2* ΔCT values, confirming the expected inverse relationship between the *TDP2* expression and ΔCT. Pre-operative TDP2 levels demonstrated a positive correlation with post-operative *TDP2* levels, with a Pearson correlation coefficient of (r = 0.353, *p* = 0.020). A further analysis indicated that pre-operative *TDP2* levels were positively correlated with log_2_ *TDP2* (r = 0.362, *p* = 0.019), thereby establishing that as the pre-operative *TDP2* expression increases, the log_2_ *TDP2* also elevates. Conversely, post-operative *TDP2* levels exhibited a strong negative correlation with log_2_ *TDP2* (r = −0.643, *p* < 0.01), which suggests a decline in log_2_ *TDP2* with higher *TDP2* levels post-operation.

**Figure 5:**
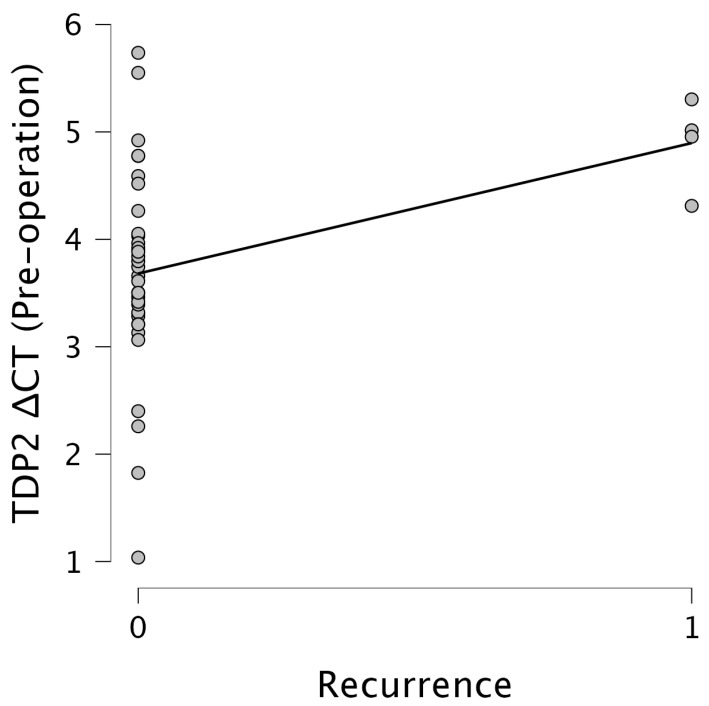
The scatter plot showing the relationship between the *TDP2* ΔCT pre-operation and recurrence. The x-axis represents the recurrence (0 = No, 1 = Yes), and the y-axis represents *TDP2* ΔCT pre-operation values. Data points cluster around 3–4 for non-recurrent cases and show a slight increase with recurrence, with a fitted line indicating a positive trend.

**Figure 6:**
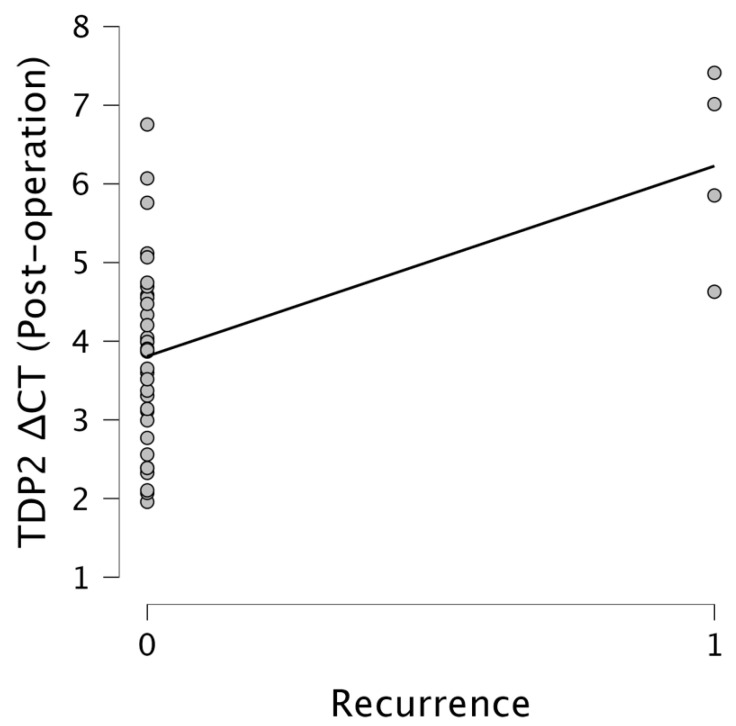
The scatter plot illustrating the relationship between the *TDP2* ΔCT post-operation and recurrence. The x-axis represents the recurrence (0 = No, 1 = Yes), and the y-axis represents *TDP2* ΔCT post-operation values. Data points are concentrated around 3–4 for non-recurrent cases, with a noticeable upward trend toward 6–7 for recurrent cases, as indicated by the fitted line.

Importantly, *TDP2* levels were significantly associated with recurrence. Pre-operative *TDP2* ΔCT values showed a moderate positive correlation with the recurrence status (r = 0.385, *p* = 0.016), while post-operative *TDP2* ΔCT values revealed a stronger correlation (r = 0.544, *p* < 0.001). This suggests that elevated post-operative *TDP2* expression levels, indicated by lower ΔCT values, are strongly associated with increased recurrence rates. Figures illustrate these relationships effectively. Figure 5 presents a scatter plot showcasing the relationship between the *TDP2* ΔCT pre-operation and the recurrence status, indicating a clustering of data points around ΔCT values of 3–4 for non-recurrent cases, with a slight upward trend observed in recurrent cases. Figure 6 emphasizes the relationship between the *TDP2* ΔCT post-operation and the recurrence, where data points primarily aggregate around 3–4 for non-recurrent cases, with a notable increase towards ΔCT values of 6–7 for recurrent cases, indicating a significant trend. The results underscore the potential of *TDP2* as a biomarker for recurrence, with lower ΔCT values reflecting higher expression levels of *TDP2* being predictive of recurrence outcomes.

### 3.5. PACS1

1. *PACS1* and *HPGD* Expression Correlations

**Figure 7:**
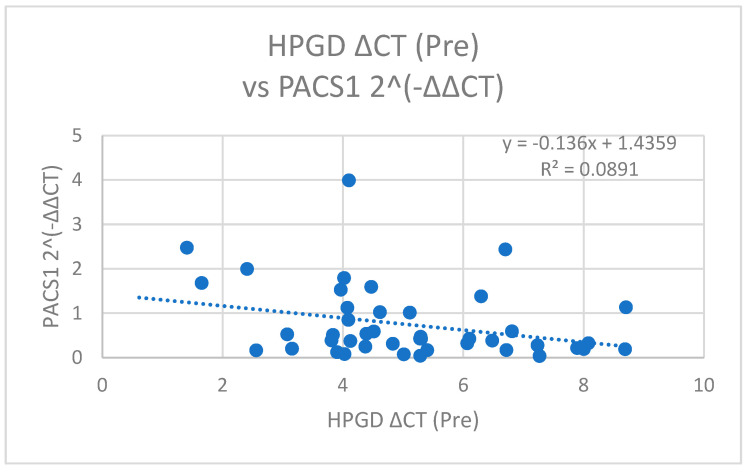
A scatter plot of *HPGD* ΔCT (Pre) versus *PACS1* 2^(−ΔΔCT) with a linear regression line (y = −0.136x + 1.4359, R^2^ = 0.0891), showing a weak negative correlation.

**Figure 8:**
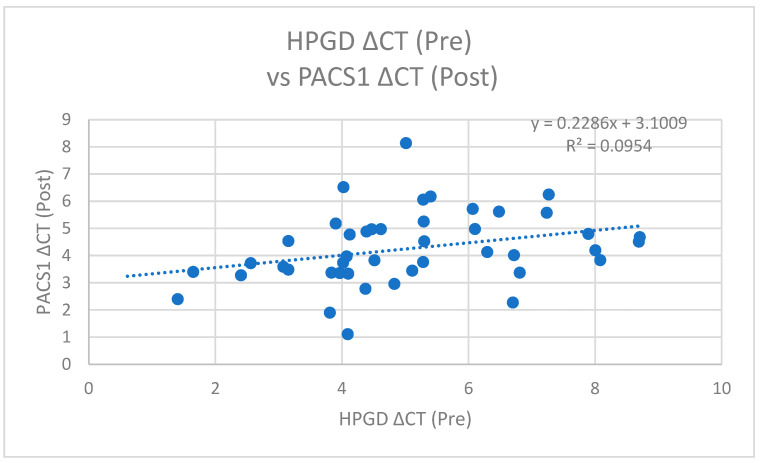
A scatter plot of *HPGD* ΔCT (Pre) versus *PACS1* ΔCT (Post) with a linear regression line (y = 0.2286x + 3.1009, R^2^ = 0.0954), showing a weak positive correlation.

We observed a significant negative correlation between *PACS1* 2^(−ΔΔCT), representing the fold change in the *PACS1* expression, and *HPGD* ΔCT values measured prior to the surgical intervention (r = −0.298, *p* = 0.049) (Figure 7). This suggests that higher expression levels of *PACS1* are associated with lower levels of *HPGD* prior to the operation, indicating a possible inverse regulatory relationship between these two biomarkers in the pre-operative setting (Figure 8).

**Figure 9:**
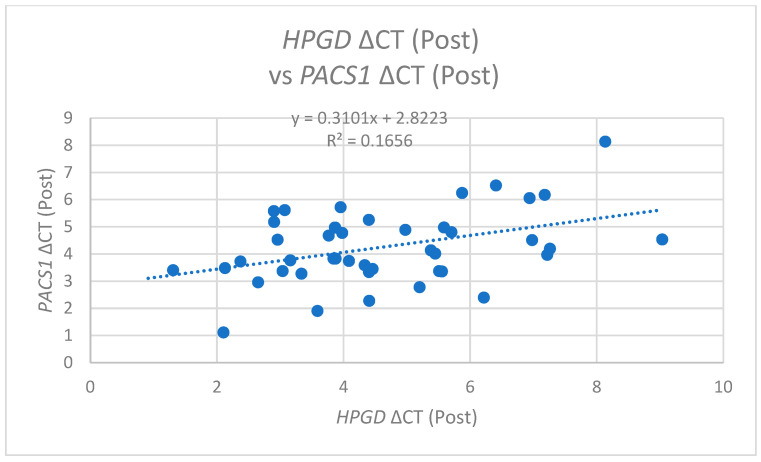
A scatter plot of *HPGD* ΔCT (Post) versus *PACS1* ΔCT (Post) with a linear regression line (y = 0.3101x + 2.8223, R^2^ = 0.1656), indicating a moderate positive correlation.

In the post-operative analysis, *PACS1* ΔCT values exhibited a positive correlation with pre-operative *HPGD* ΔCT (r = 0.309, *p* = 0.041) (Figure 8) and also with post-operative *HPGD* levels (r = 0.407, *p* = 0.006) (Figure 9). This suggests that following the surgical intervention, increases in the *PACS1* expression may facilitate or promote the expression of *HPGD*, further underscoring the dynamic interplay between these molecules during operations.

2. *PACS1* and Survival Time

**Figure 10:**
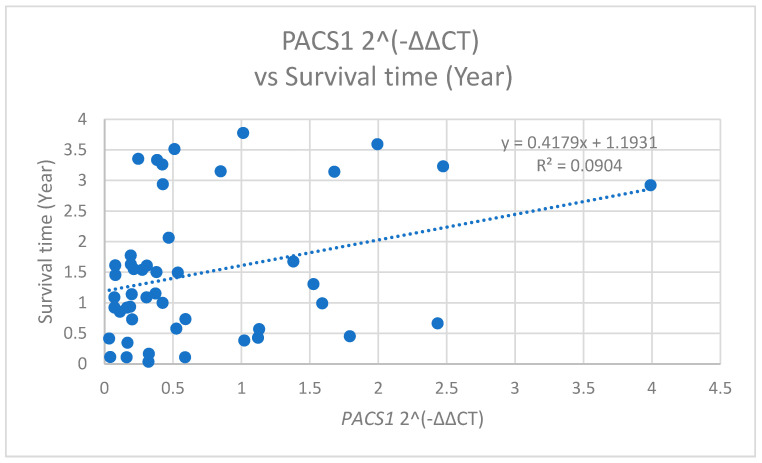
The scatter plot of *PACS1* 2^(−ΔΔCT) versus the survival time (years) with a linear regression line (y = 0.4179x + 1.9131, R^2^ = 0.0904), indicating a weak positive correlation between the *PACS1* relative expression and survival time.

The analysis of *PACS1* expression levels in relation to the patient survival time unveiled significant correlations. Specifically, *PACS1* 2^(−ΔΔCT) values was positively correlated with the survival time, measured in years (r = 0.301, *p* = 0.038) (Figure 10). This indicates that a higher *PACS1* expression is associated with shorter survival times.

**Figure 11:**
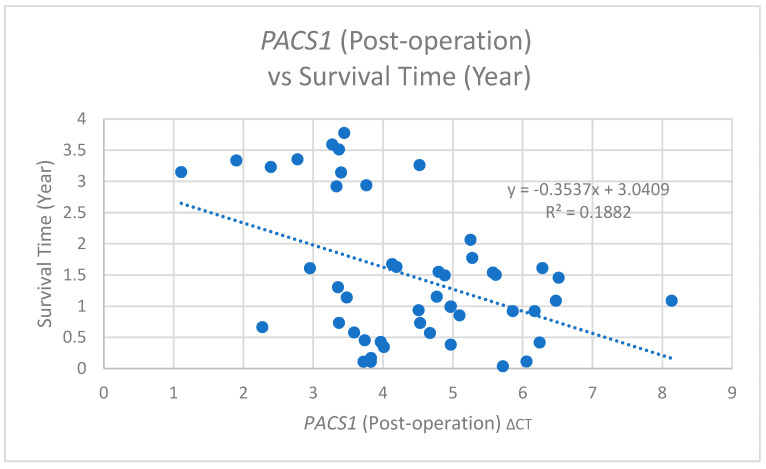
A scatter plot of *PACS1* (post-operation) ΔCT versus the survival time (years) with a linear regression line (y = −0.3537x + 3.0409, R^2^ = 0.1882), showing a moderate negative correlation between post-operative PACS1 ΔCT and the survival time.

Conversely, the *PACS1* ΔCT measured post-operation demonstrated a significant negative correlation with the survival duration (r = −0.434, *p* = 0.002) (Figure 11). The contrasting natures of these correlations suggest that the timing of the *PACS1* measurement, whether pre- or post-operation, may influence its prognostic capabilities, warranting a further exploration into the mechanisms through which *PACS1* impacts patient survival.

3. PACS1 and Clinical Parameters

We further assessed the relationship of *PACS1* ΔCT (pre-operation) with various clinical parameters, including gender and age. A significant positive correlation was found between the *PACS1* ΔCT (pre) and gender (r = 0.296, *p* = 0.039), as well as between *PACS1* ΔCT (pre) and age (r = 0.311, *p* = 0.030). These findings indicate that demographic factors may influence PACS1 expression levels, suggesting a need for the consideration of these parameters when evaluating *PACS1* as a potential biomarker.

4. PACS1 Expression Variability

Moreover, a strong positive correlation was observed between the *PACS1* 2^(−ΔΔCT) and *PACS1* ΔCT (pre) values (r = 0.563, *p* < 0.001), indicating a robust relationship between these two measures and supporting the reliability of PACS1 as a biomarker. However, *PACS1* 2^(−ΔΔCT) values showed a significant negative correlation with post-operative *PACS1* ΔCT (r = −0.470, *p* < 0.001). This suggests that significant alterations in the *PACS1* expression occur post-operation, potentially reflecting the therapeutic effect of the intervention on biomarker levels.

### 3.6. Clinical Variables

Age was negatively correlated with the survival in years (r = −0.283, *p* = 0.044), suggesting that older patients had shorter survival times. The Duke stage was strongly correlated with pathological staging variables, including pN (r = 0.531, *p* < 0.001), pM (r = 0.756, *p* < 0.001), and pT (r = 0.365, *p* = 0.008), indicating a robust association between the tumor staging and disease severity.

## 4. Discussion

CRC persists as one of the most significant public health challenges globally, characterized by alarming incidence and mortality rates. The findings of our study further emphasize the urgent need to improve prognostic assessments and therapeutic strategies to mitigate the recurrence rates and late-stage diagnoses characteristic of this malignancy. The validation of our three cfRNA biomarkers, *HPGD*, *PACS1* and *TDP2,* reveals a promising approach for the early identification of MRD in CRC patients.

Our results indicate that pre-operative and post-operative expression levels of *HPGD* correlate significantly with survival outcomes, mirroring prior findings that suggested its potential as a prognostic biomarker in CRC. Higher expression levels of *HPGD* have been associated with better survival rates in this study, aligning with observations regarding tumor dynamics post-operation from our previous study [7], which emphasize that cfRNA profiles could reflect shifts in the tumor burden and treatment responses, enhancing their utility in clinical settings. These findings resonate with prior studies suggesting *HPGD*’s role as a prognostic biomarker in CRC, where higher levels of this enzyme are associated with improved survival rates of the other cancer types, such as breast cancer [9] and prostate cancer [10]. Moreover, the recent work by Zhai et al. concurs with our observations regarding the prognostic significance of *HPGD*, highlighting its regulatory effects in cancer progression and immune environment monitoring (Zhai et al., 2024). Their investigations suggest that *HPGD* plays an essential role in determining disease outcomes, further establishing the link between this biomarker and survival [11]. Similarly, Padilla et al. highlight high *HPGD* expression levels across various malignancies, proposing its function as a tumor suppressor linked to better prognostic outcomes [9]. Further reinforcing the clinical relevance of cfRNAs, studies have shown that HPGD’s involvement in the prostaglandin metabolic pathway is crucial for understanding its impact on tumor growth and the inflammatory microenvironment surrounding cancers [12]. Elevated COX-2 activity has been previously associated with decreased HPGD levels, suggesting a reciprocal relationship that can drive tumor progression [13]. This interplay may elucidate why monitoring HPGD levels in patients could provide critical insights into treatment efficacy and patient management strategies.

The findings of our study also highlight the critical relationship between *PACS1* and *HPGD* expression levels, which may serve as prognostic biomarkers in the context of CRC. Our analysis shows a significant correlation between *PACS1* and *HPGD* expression levels post-operation, suggesting that PACS1 may act to regulate the *HPGD* expression in response to changes within the tumor microenvironment. These results align with our previous study (Jin et al., 2023), where we demonstrated that expression profiles of *HPGD*, *PACS1*, and *TDP2* were consistent across cfRNAs and tissue samples, underscoring the potential of these genes as robust biomarkers for CRC [7]. The interplay between these two biomarkers could offer insights into their collective roles in influencing tumor dynamics and the patient prognosis. Specifically, *PACS1* has been implicated in apoptosis through its regulation of BAX and BAK, highlighting its potential impact on cancer cell survival and treatment responses [14,15]. Furthermore, our findings underscore the necessity of understanding demographic influences, such as age and gender, on biomarker expression levels, as these factors may play a pivotal role in patient-specific responses to therapy. Integrating demographic influences into clinical practice is crucial; however, the existing references do not adequately support the claims regarding their impact on the *PACS1* expression and CRC outcomes, indicating a need for more direct evidence. The integration of *PACS1* and *HPGD* expression profiles in clinical settings could facilitate personalized therapeutic strategies, optimizing treatment regimens based on individual biomarker profiles. Overall, these insights pave the way for future investigations into the mechanistic underpinnings of *PACS1* and *HPGD* interactions, potentially reinforcing their roles as key players in the CRC prognosis and management.

Our findings also extend the understanding of TDP2’s role in cancer progression. Elevated levels of *TDP2* were linked with an increased risk of recurrence, reinforcing the significance of this gene in DNA repair mechanisms and its potential involvement in therapeutic responses. As highlighted by Manguso et al., 2024, *TDP2*, along with other key genes, constitutes a vital prognostic indicator, reinforcing its utility in non-invasive diagnostics [16]. *TDP2* is crucial for resolving DNA double-strand breaks involving topoisomerase II (TOP2); its deficiency has been linked to genomic instability and heightened tumorigenesis [16,17]. Additionally, *TDP2*’s interactions within DNA repair pathways and oncogenic signaling further establish its role in cancer progression and therapeutic responses, making it a promising target for personalized cancer treatment approaches [17]. Consequently, integrating a *TDP2* analysis with cfRNA profiling could enhance prognostic capabilities and inform tailored therapeutic strategies for CRC management.

The integration of biomarkers such as *HPGD*, *PACS1*, and *TDP2* into clinical diagnostics holds significant promise for transforming current practices surrounding MRD detection in CRC. Traditional diagnostic methods often struggle with limitations in sensitivity and specificity, which necessitates the development of more reliable, non-invasive alternatives. The findings from this study support the potential of a blood-based test that measures levels of cfRNAs for identifying patients at an elevated risk of recurrence significantly earlier than conventional methods permit. Future research should concentrate on expanding the cohort size and examining the long-term prognostic implications of diverse cfRNA profiles over multiple post-operative time points to further validate the robustness of our findings.

Moreover, it is crucial to evaluate the applicability of those cfRNA biomarkers beyond the predominantly Chinese cohorts in current studies. The genetic variability among different ethnicities can significantly influence the expression and reliability of cfRNAs as biomarkers [18]. Studies have suggested that cancer-specific cfRNAs exhibit expression patterns that are not universally consistent across various demographic groups [18,19]. Additionally, different validation studies indicate the importance of having a diverse reference dataset to evaluate cfRNA biomarkers and address potential biases that may arise from tissue or cell-type specificity [20]. Hence, the exploration of these cfRNA biomarkers within multiethnic cohorts is essential to enhance the reliability in clinical settings and support personalized medicine approaches in CRC management [18].

The current study has several limitations that should be addressed to enhance its overall impact. Specifically, a more thorough examination of the biological roles and mechanisms governing the differential regulation of *HPGD*, *PACS1*, and *TDP2* in CRC is necessary, as this could elucidate the pathways through which these genes contribute to tumor biology, particularly in light of the conflicting *TDP2* results. Furthermore, while the initial cohort of 52 patients offers valuable insights, a larger sample size achieved through multicentric studies would improve the statistical power and generalizability of our findings. Additionally, it is important to address the technical aspects of the qPCR calibration, quality control measures, and normalization strategies to ensure a robust data interpretation. Future investigations should also implement a longitudinal design that assesses the prognostic relevance of these biomarkers across multiple post-operative time points, thereby providing a more comprehensive understanding of their kinetic profiles.

## 5. Conclusions

This study underscores the potential of cfRNAs as non-invasive biomarkers in the context of CRC prognosis. The significant upregulation of *HPGD* and *PACS1*, alongside the contrasting variable expression of *TDP2*, enriches our understanding of the cfRNA landscape and its association with clinical outcomes. Specifically, the finding that lower levels of *HPGD* correlate with diminished survival rates positions it as a critical negative prognostic indicator, while a high *TDP2* expression is linked to increased recurrence risks, underlining its value in disease monitoring. The complexities revealed in the expression patterns of these cfRNAs highlight their intricate roles within the CRC pathology and their clinical applicability. Therefore, the findings advocate for further investigations into the prognostic capabilities of *PACS1* and emphasize the necessity of integrated cfRNA assessments, which may enhance patient stratification and therapeutic decision-making in colorectal cancer management, thereby influencing future molecular oncology approaches.

## Figures and Tables

**Table 1:** Commercially available Taqman gene expression assays.

Gene Name	Assay ID	Target	Primer Condition
*HPGD*	Hs00960591_m1	Hydroxyprostaglandin Dehydrogenase	Annealing: 60 °C
*PACS1*	Hs01555555_g1	Phosphofurin Acidic Cluster Sorting Protein 1	Annealing: 60 °C
*TDP2*	Hs01099017_m1	Tyrosyl-DNA Phosphodiesterase 2	Annealing: 60 °C
*GAPDH*	Hs02786624_g1	Glyceraldehyde-3-Phosphate Dehydrogenase	Annealing: 60 °C

**Table 2:** Comparing the Taqman qPCR and RNA-seq data for the cfRNA expression of *HPGD*, *PACS1*, and *TDP2* in CRC patients, building on Jt al. (2023) [7] by sing the one-sample *t*-test.

	t	df	*p*	Taqman Regulation	RNA-Seq Regulation from Jin 2023 [7]
Log_2_ *HPGD* 2^(−ΔΔCT)	2.254	43	0.029	Upregulate	Upregulate
Log_2_ *TDP2* 2^(−ΔΔCT)	−0.398	41	0.693	Downregulate	Upregulate
Log_2_ *PACS1* 2^(−ΔΔCT)	−5.938	48	<0.001	Downregulate	Downregulate

## Data Availability

Data is contained within the article.
